# 

**Published:** 1876-04

**Authors:** 


### CONTENTS.

PAGE.

**CONTRIBUTIONS.**

The Authority. BEN. H. PRATT....113
Doctors. DR. NED....115
Prophylaxis of Typhoid Fever. H....117
Foundered. P. H. HAYES, M. D....118

**MEDICAL ITEMS AND NEWS.**

Local Anæsthesia—Thread Worms—Offensive discharge from Cancer—To make Court Plaster Adhere—Hiccough—Carbolic Acid Poisoning—Carbolic Acid as an Anthelmintic—Post-Partum Hemorrhage—Palpitation of Heart—Facial Neuralgia—Neuralgia—Blistering Substance—Cancer—Carious Teeth—Topical Application in Painful Dentition—Jaborandi and Atropia—Chronic Hoarseness--Hydrocele--Russian Cure for Drunkenness—Itch—Fissure of the Anus—Malaria—Ergot, New Preparation of--Croupous Pneumonia—Eczema in Children—Scarlatina—Calabar Bean—Habitual Constipation—Pumpkin Seeds as Anthelmintics—Salicylic Acid in Obstetric Practice—Acute Rheumatism—Chorea—Wounds—Use of Apocynum Cannabinum in Dropsies—Salicylic Acid in Otorrhoea—Chorea—Discoloration by Ox. Silver—Intestinal Obstructions—Estimation of Uric Acid—Gelsemium—Chloral as a Surgical Dressing—Rheumatism, Epilepsy—Scarlatina—Diabetes—"Certain" Cure for Rheumatism—Night Sweats of Phthisis—Hæmoptysis—Quinia and Morphia....120 to 129

**OUR CORRESPONDENCE.**

Fourteen Letters with Replies....129 to 133
"The Great English Physician."....132

**EDITORIAL.**

Infants Eyes....133
A German's Experience with Quacks....134
Flour Making....135

**EDITORIAL CHIT-CHAT.**

Our Correspondence—Is it You?—Returned H.—"Elmira Medical and Surgical Association"—In Arrears—Poor Robin--Hartz--Floral Decorations—Tricks o' Trade—Wise Doctors—Busy and Dirty Scranton—Reliable Medicines—"How to Acquire and Preserve Health"—Trouting—Ride on a Locomotive—Elmira Water Cure—Florida—That Royal Train—Cosmoline—Minature Mine—Centennial Times—Pigeon Shooting—Pocket Gymnasium—Receipts....136 to 140

